# Dataset on the knowledge, attitudes and practices of university students towards antibiotics

**DOI:** 10.1016/j.dib.2018.06.090

**Published:** 2018-06-30

**Authors:** Olayemi O. Ayepola, Olabode A. Onile-Ere, Oluwatobi E. Shodeko, Fiyinfolouwa A. Akinsiku, Percy E. Ani, Louis Egwari

**Affiliations:** Department of Biological Sciences, Covenant university, Ogun State, Nigeria

## Abstract

Antibiotic resistance is a major public health issue globally fuelled largely by its misuse. Controlling this problem would require an understanding of the levels of awareness of the population towards antibiotics. The data presented here was obtained from undergraduate students attending a Nigerian University in the first three months of the year 2016. The data is stratified by such demographic variables as age, sex and level of study. It contains information about the knowledge, and predispositions of participants to antibiotics and antibiotic resistance. Preliminary descriptive statistics are presented in the tables and figures herewith. Data was analysed using SPSS-20 and is available for reuse in the native SPSS format. In concluding, this data can be used to model the determinants of antibiotic knowledge among students.

**Specifications Table**

**Table:** 

Subject area	*Pharmaceutical Microbiology*
More specific subject area	*Antibiotic Stewardship, Antibiotic Resistance*
Type of data	*Table and figure*
How data was acquired	*Cross-Sectional survey*
Data format	*Raw and analyzed*
Experimental factors	*Data obtained from students in a cross-sectional study*
Experimental features	*Structured Questionnaires were administered to students of a university to assess their predisposition towards antibiotics and antibiotic resistance. Descriptive statistics, frequency distributions and Chi-square statistic were computed to determine the predictors of antibiotic knowledge.*
Data source location	*Ado-Odo, Ota Ogun State Nigeria*
Data accessibility	*Data is publicly available in Mendeley Data* DOI: 10.17632/xh75bp2dmy.1.

**Value of the data**

• The dataset presented here reports the attitudes of university students towards antibiotics and antibiotic resistance as such it could, in tandem with other datasets, be used to model predictors for antibiotic stewardship.
• The dataset could be useful in designing targeted intervention programs in the study area.
• The data alongside the questionnaire provided here could serve as a benchmark for other researchers who would conduct similar research.

## 1 Data

The data described here was collected, using a structured questionnaire, between January and March 2016 from undergraduate students attending Covenant University, Ogun State Nigeria. A 35-item questionnaire was developed from existing studies [1], [2], [3], [4], [5]. The self-administered questionnaire was designed to obtain demographic information of participants, assess patterns of antibiotic usage, perceptions and knowledge of antibiotics among students. The data contains demographic variables for clustering study participants alongside indicators of antibiotic knowledge, perception and usage. To make data more granular, we classified respondents into 2 broad groups; Science and Non-Science. Respondents from the College of Science and Technology (CST) and College of Engineering (CoE) were classified as Science while respondents from College of Business Studies (CBS) and College of Developmental Studies (CDS) were classified as Non-Science. A knowledge score was computed from a subset 10 questions with respondents given 1 point for a correct answer and no points for a wrong answer. Persons scoring 6 and above were considered to have good knowledge. The descriptive analysis presented here is divided into three sections; Summary of study participants, patterns of antibiotic usage and Knowledge of antibiotics.

### 1.1 Summary of study participants

**Fig. 1:**
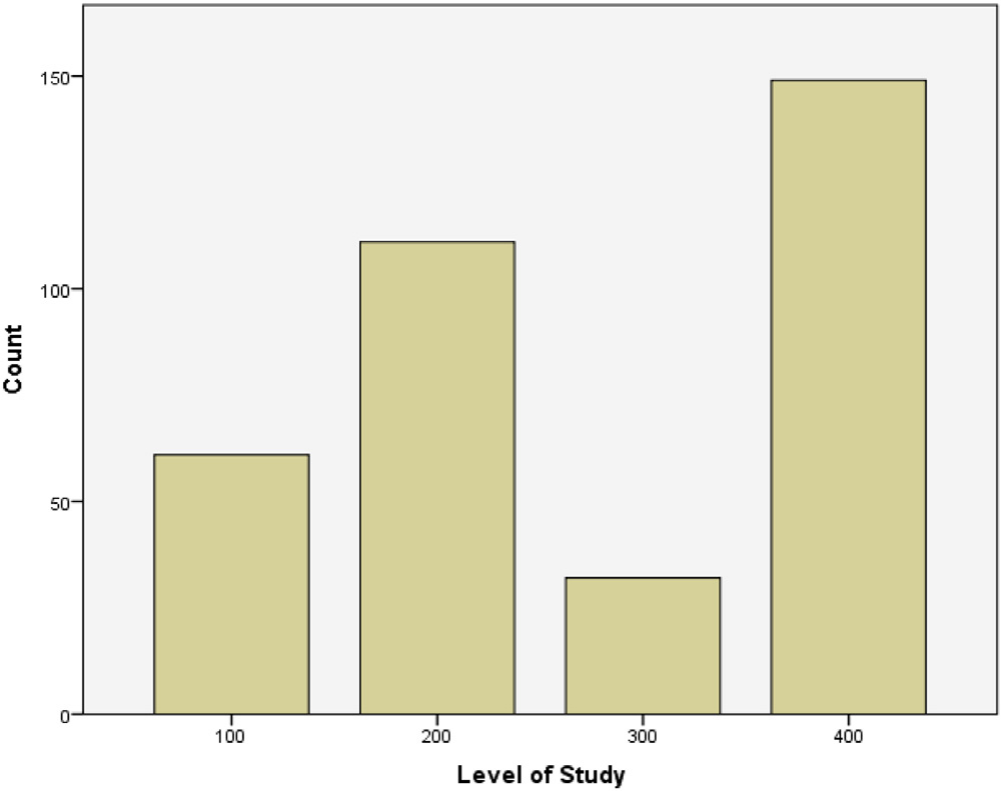
Bar chart showing the distribution of students across the different levels.

**Fig. 2:**
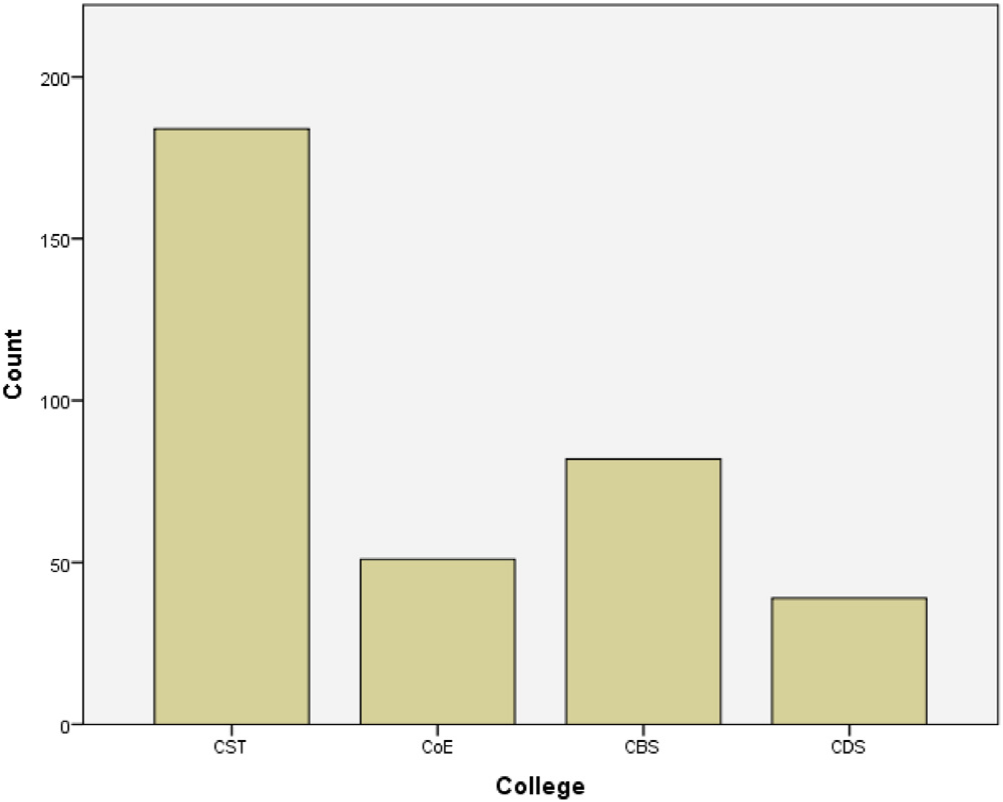
Bar chart showing the distribution of students across colleges.

**Fig. 3:**
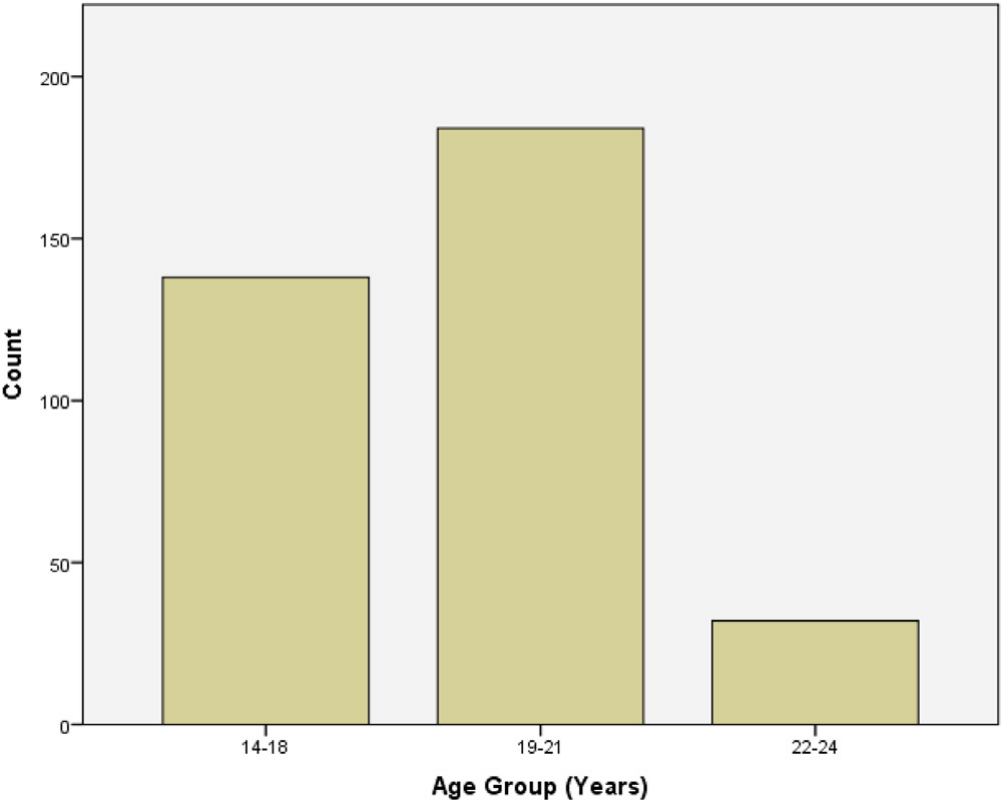
Bar chart showing the distribution of age groups.

See Table 1 and Fig. 1, Fig. 2, Fig. 3.

**Table 1:** Summary of study participants.

		Count	Column *N* %
College	CST	184	51.7
	CoE	51	14.3
	CBS	82	23.0
	CDS	39	11.0
Level	100	61	17.3
	200	111	31.4
	300	32	9.1
	400	114	32.3
	500	35	9.9
Age group	14–18	138	39.0
	19–21	184	52.0
	22–24	32	9.0
Sex	Male	152	42.8
	Female	203	57.2

CST – College of science and technology.

CoE – College of engineering.

CBS – College of business studies.

CDS – College of developmental studies.

### 1.2 Patterns of antibiotic usage among participants

**Fig. 4:**
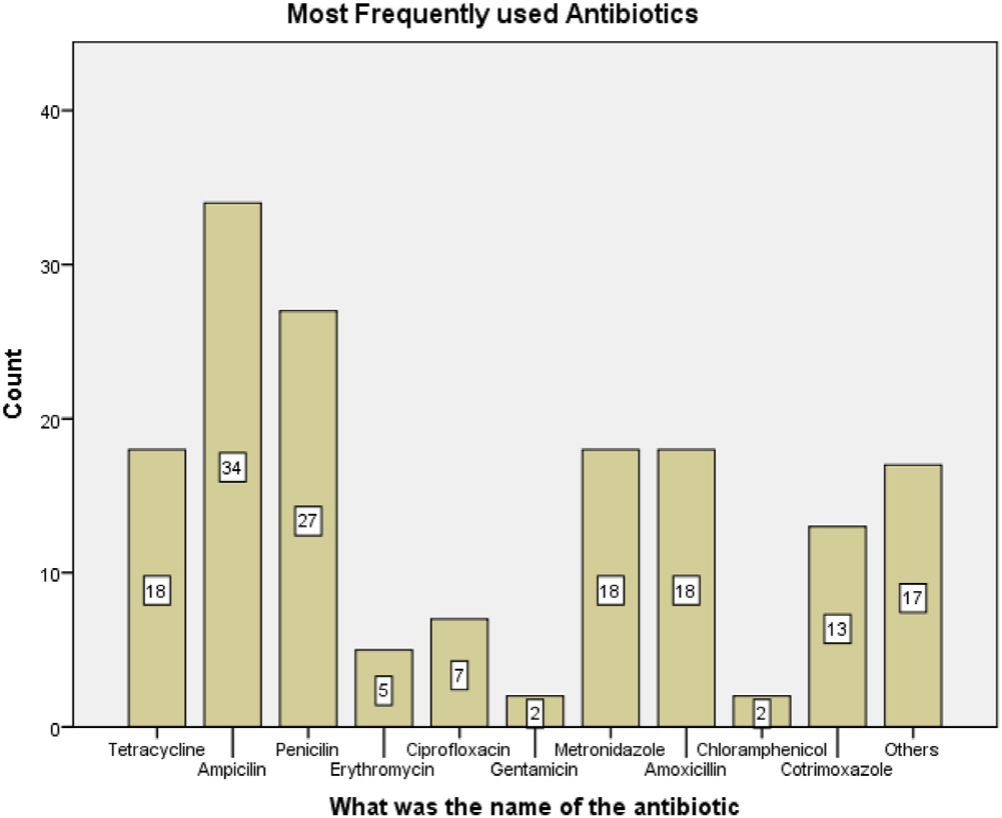
Frequency distribution of antibiotic usage.

**Fig. 5:**
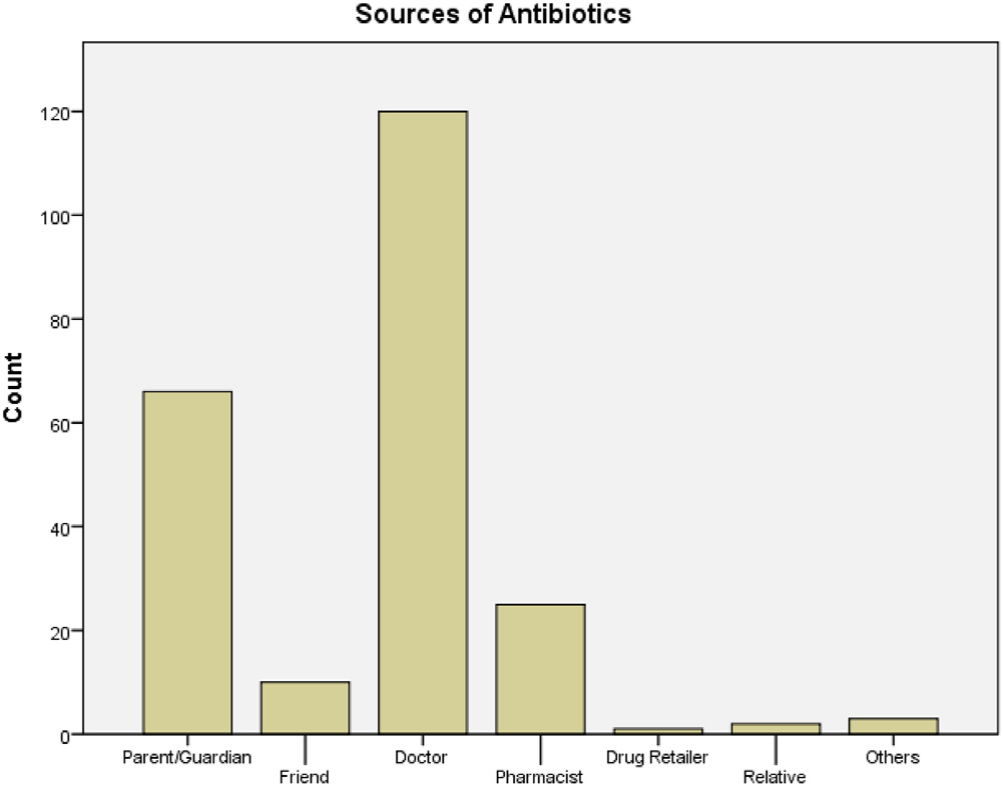
Frequency distribution of the different sources of antibiotics.

See Tables 2 and 3 and Figs. 4 and 5.

**Table 2:** Patterns of antibiotic usage among study participants I.

	Yes		No	
	Count	Row *N* %	Count	Row *N* %
Have you taken Antibiotics in the past six (6) months?	214	60.6	139	39.4
Did You Adhere Strictly to the dosage instructions	176	75.2	58	24.8
Do you think its important to complete the drug dosage, even if all symptoms are gone?	225	73.3	82	26.7
Do you always complete your dose as prescribed by the physician	138	42.2	189	57.8
Do you keep leftover drugs for future use?	189	56.9	143	43.1
Are you aware that the improper use of antibiotics could be harmful?	252	74.8	85	25.2

**Table 3:** Patterns of antibiotic usage among study participants II.

	Always/Often		Rarely/Sometimes		Never	
	Count	Row *N* %	Count	Row *N* %	Count	Row *N* %
Have you ever used antibiotics without a doctor׳s prescription	218	64.5	113	33.4	7	2.1
If the doctors refused to prescribe antibiotics for you, would you insist on the doctor doing so?	63	18.5	250	73.5	27	7.9

### 1.3 Knowledge of antibiotics

**Fig. 6:**
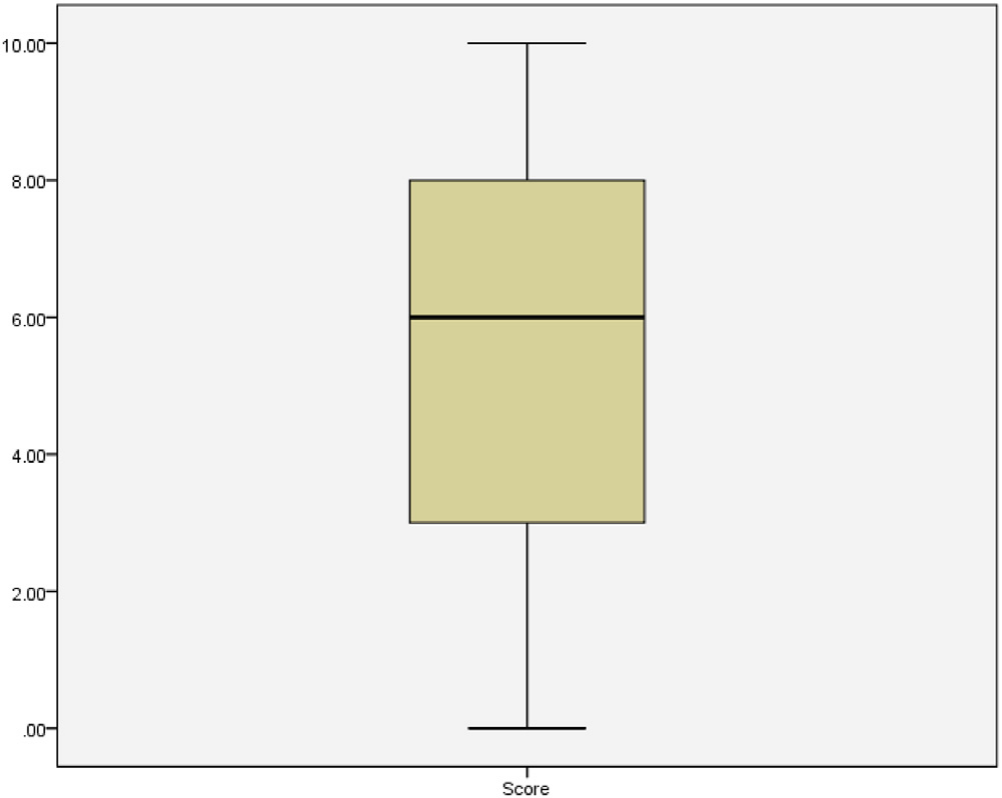
Box plot of knowledge scores.

**Fig. 7:**
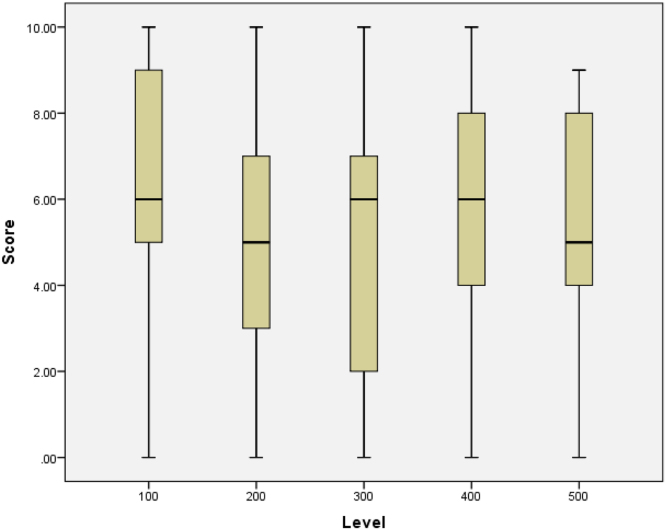
Box plot of knowledge scores by level of study.

**Fig. 8:**
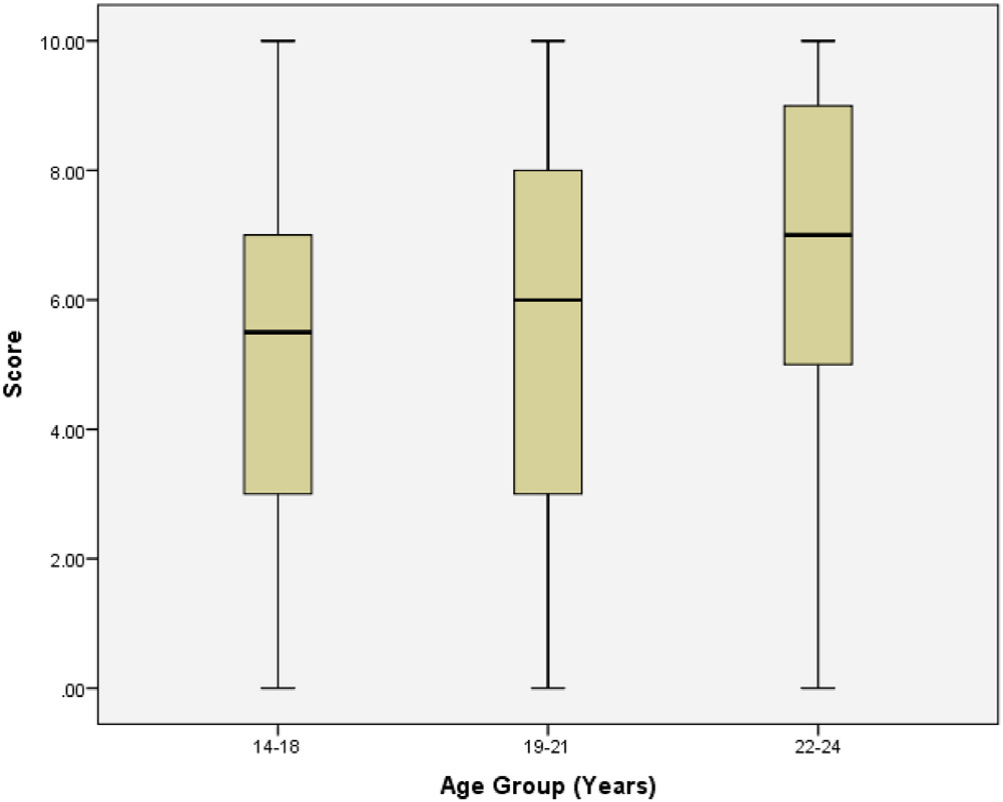
Box plot of knowledge scores by age group.

**Fig. 9:**
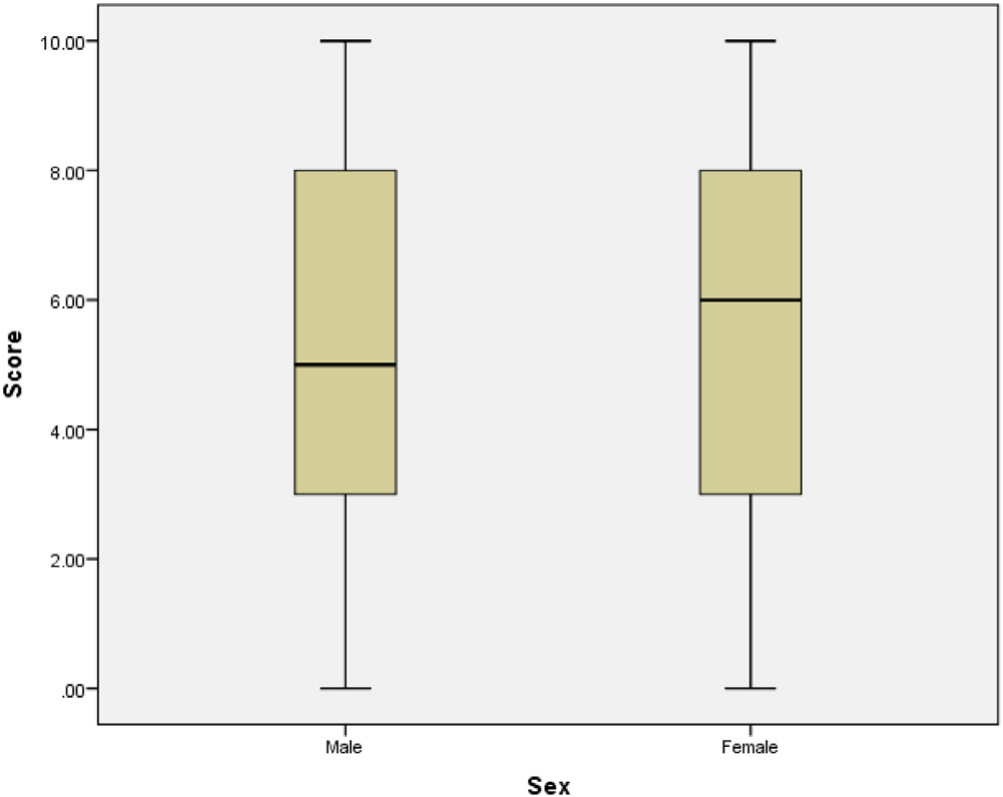
Box plot of knowledge scores by sex.

**Fig. 10:**
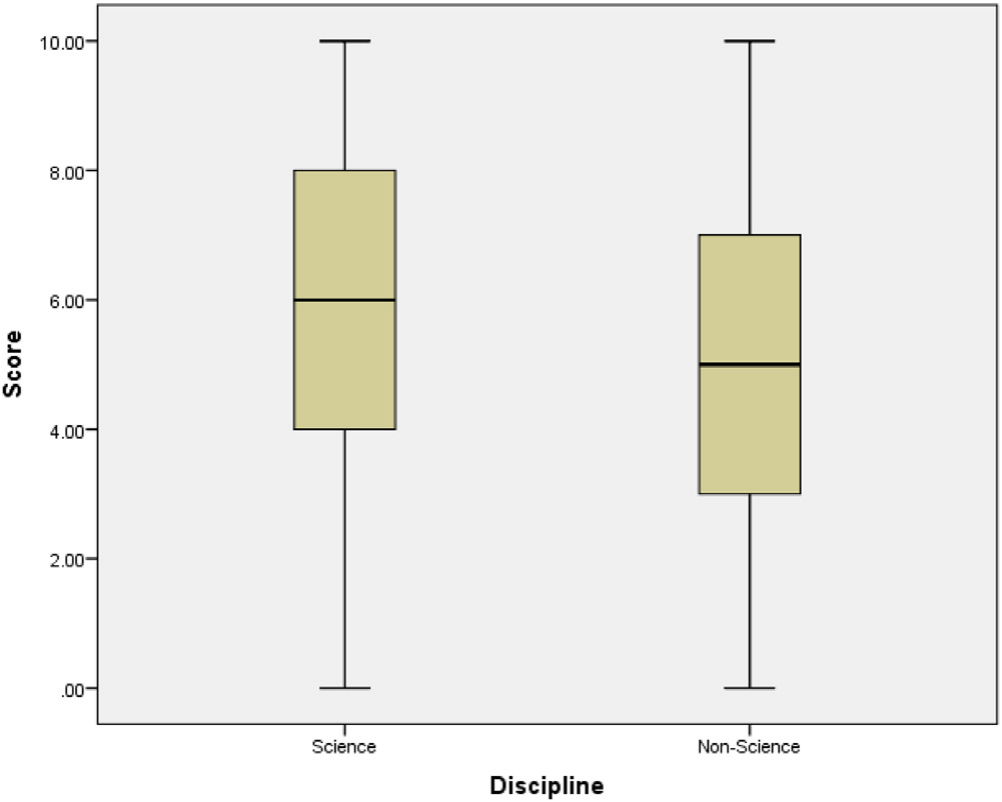
Box plot of knowledge scores by discipline.

See Table 4, Table 5, Table 6, Table 7, Table 8, Table 9 and Fig. 6, Fig. 7, Fig. 8, Fig. 9, Fig. 10.

**Table 4:** Summary statistics for knowledge score.

			Statistic	Std. error
Knowledge score	Mean		5.5084	0.14280
	95% Confidence Interval for Mean	Lower Bound	5.2276	
		Upper Bound	5.7893	
	5% Trimmed Mean		5.5468	
	Median		6.0000	
	Variance		7.259	
	Std. Deviation		2.69427	
	Minimum		0.00	
	Maximum		10.00	
	Range		10.00	
	Interquartile Range		5.00	
	Skewness		−0.217	0.129
	Kurtosis		−0.895	0.258

**Table 5:** Summary statistics of knowledge scores by level of study.

			Level									
			100		200		300		400		500	
			Statistic	Std. Error	Statistic	Std. Error	Statistic	Std. Error	Statistic	Std. Error	Statistic	Std. Error
Score	Mean		6.4754	0.29532	4.7636	0.25240	4.9688	0.50298	5.9649	0.25442	5.4000	0.44571
	95% Confidence Interval for Mean	Lower Bound	5.8847		4.2634		3.9429		5.4609		4.4942	
		Upper Bound	7.0661		5.2639		5.9946		6.4690		6.3058	
	5% Trimmed Mean		6.5638		4.7677		4.9653		6.0458		5.5000	
	Median		6.0000		5.0000		6.0000		6.0000		5.0000	
	Variance		5.320		7.008		8.096		7.379		6.953	
	Std. Deviation		2.30656		2.64723		2.84531		2.71648		2.63684	
	Minimum		0.00		0.00		0.00		0.00		0.00	
	Maximum		10.00		10.00		10.00		10.00		9.00	
	Range		10.00		10.00		10.00		10.00		9.00	
	Interquartile Range		4.00		4.25		5.00		4.00		4.00	
	Skewness		−0.397	0.306	0.049	0.230	−0.047	0.414	−0.375	0.226	−0.473	0.398
	Kurtosis		−0.054	0.604	−1.032	0.457	−1.153	0.809	−0.745	0.449	−0.834	0.778

**Table 6:** Knowledge by level of study.

			Level					Total
			100	200	300	400	500	
Knowledge	Poor Knowledge	Count	22	69	14	46	18	169
		% within Knowledge	13.0%	40.8%	8.3%	27.2%	10.7%	100.0%
	Good Knowledge	Count	39	42	18	68	17	184
		% within Knowledge	21.2%	22.8%	9.8%	37.0%	9.2%	100.0%
Total		Count	61	111	32	114	35	353
		% within Knowledge	17.3%	31.4%	9.1%	32.3%	9.9%	100.0%

**Table 7:** Summary statistics of knowledge scores by age group.

			Age Group					
			14–18		19–21		22–24	
			Statistic	Std. Error	Statistic	Std. Error	Statistic	Std. Error
Score	Mean		5.3768	0.22865	5.4645	0.20025	6.6129	0.45361
	95% Confidence Interval for Mean	Lower Bound	4.9247		5.0694		5.6865	
		Upper Bound	5.8289		5.8596		7.5393	
	5% Trimmed Mean		5.4267		5.4970		6.7724	
	Median		5.5000		6.0000		7.0000	
	Variance		7.215		7.338		6.378	
	Std. Deviation		2.68601		2.70888		2.52557	
	Minimum		0.00		0.00		0.00	
	Maximum		10.00		10.00		10.00	
	Range		10.00		10.00		10.00	
	Interquartile Range		4.00		5.00		4.00	
	Skewness		−0.204	0.206	−0.195	0.180	−0.699	0.421
	Kurtosis		−0.903	0.410	−0.959	0.357	0.508	0.821

**Table 8:** Summary statistics of knowledge scores by sex.

			Sex			
			Male		Female	
			Statistic	Std. Error	Statistic	Std. Error
	Mean		5.2980	0.21424	5.7065	0.19328
	95% Confidence Interval for Mean	Lower Bound	4.8747		5.3253	
		Upper Bound	5.7213		6.0876	
	5% Trimmed Mean		5.3164		5.7681	
	Median		5.0000		6.0000	
	Variance		6.931		7.508	
Score	Std. Deviation		2.63260		2.74015	
	Minimum		0.00		0.00	
	Maximum		10.00		10.00	
	Range		10.00		10.00	
	Interquartile Range		5.00		5.00	
	Skewness		−0.113	0.197	−0.335	0.172
	Kurtosis		−0.889	0.392	−0.838	0.341

**Table 9:** Summary statistics of knowledge scores by discipline.

			Discipline			
			Science		Non-Science	
			Statistic	Std. Error	Statistic	Std. Error
Score	Mean		5.7489	0.17901	5.0413	0.23100
	95% Confidence Interval for Mean	Lower Bound	5.3963		4.5840	
		Upper Bound	6.1016		5.4987	
	5% Trimmed Mean		5.8097		5.0826	
	Median		6.0000		5.0000	
	Variance		7.531		6.457	
	Std. Deviation		2.74421		2.54099	
	Minimum		0.00		0.00	
	Maximum		10.00		10.00	
	Range		10.00		10.00	
	Interquartile Range		4.00		4.00	
	Skewness		−0.273	0.159	−0.190	0.220
	Kurtosis		−0.893	0.316	−0.895	0.437

## 2 Experimental design, materials and methods

This study was carried out in Covenant University, Ota, Ogun State Nigeria. Covenant University offers a wide variety of courses, cutting across many disciplines and has a student population of about 8000 undergraduate and postgraduate students. The responses were collected from undergraduate students. Random selection method was used to recruit students into the study. Responses obtained were entered into SPSS-20. Descriptive statistics of the data is presented here.
